# Enhanced CD4^+^ and CD8^+^ T cell infiltrate within convex hull defined pancreatic islet borders as autoimmune diabetes progresses

**DOI:** 10.1038/s41598-021-96327-2

**Published:** 2021-08-25

**Authors:** Alexander J. Dwyer, Jacob M. Ritz, Jason S. Mitchell, Tijana Martinov, Mohannad Alkhatib, Nubia Silva, Christopher G. Tucker, Brian T. Fife

**Affiliations:** 1 Center for Immunology, Department of Medicine, Division of Rheumatic and Autoimmune Diseases, University of Minnesota Medical School, 2101 6th St SE, Wallin Medical Biosciences Building, 3-146, Minneapolis, MN 55455 USA; 2 School of Physics and Astronomy, University of Minnesota, Twin Cities, Minneapolis, MN USA; 3 Center for Immunology, Department of Laboratory Medicine and Pathology, University of Minnesota Medical School, Minneapolis, MN USA; 4 Clinical Research Division, Program in Immunology, Fred Hutchinson Cancer Research Center, Seattle, WA USA

**Keywords:** Autoimmunity, Imaging the immune system

## Abstract

The notion that T cell insulitis increases as type 1 diabetes (T1D) develops is unsurprising, however, the quantitative analysis of CD4^+^ and CD8^+^ T cells within the islet mass is complex and limited with standard approaches. Optical microscopy is an important and widely used method to evaluate immune cell infiltration into pancreatic islets of Langerhans for the study of disease progression or therapeutic efficacy in murine T1D. However, the accuracy of this approach is often limited by subjective and potentially biased qualitative assessment of immune cell subsets. In addition, attempts at quantitative measurements require significant time for manual analysis and often involve sophisticated and expensive imaging software. In this study, we developed and illustrate here a streamlined analytical strategy for the rapid, automated and unbiased investigation of islet area and immune cell infiltration within (insulitis) and around (peri-insulitis) pancreatic islets. To this end, we demonstrate swift and accurate detection of islet borders by modeling cross-sectional islet areas with convex polygons (convex hulls) surrounding islet-associated insulin-producing β cell and glucagon-producing α cell fluorescent signals. To accomplish this, we used a macro produced with the freeware software ImageJ equipped with the Fiji Is Just ImageJ (FIJI) image processing package. Our image analysis procedure allows for direct quantification and statistical determination of islet area and infiltration in a reproducible manner, with location-specific data that more accurately reflect islet areas as insulitis proceeds throughout T1D. Using this approach, we quantified the islet area infiltrated with CD4^+^ and CD8^+^ T cells allowing statistical comparison between different age groups of non-obese diabetic (NOD) mice progressing towards T1D. We found significantly more CD4^+^ and CD8^+^ T cells infiltrating the convex hull-defined islet mass of 13-week-old non-diabetic and 17-week-old diabetic NOD mice compared to 4-week-old NOD mice. We also determined a significant and measurable loss of islet mass in mice that developed T1D. This approach will be helpful for the location-dependent quantitative calculation of islet mass and cellular infiltration during T1D pathogenesis and can be combined with other markers of inflammation or activation in future studies.

## Introduction

### T cells in type 1 diabetes

Type 1 diabetes (T1D) is an autoimmune disease characterized by T cell-mediated destruction of the insulin-producing β cells in the pancreatic islets of Langerhans^1^. Both CD4^+^ and CD8^+^ T cells are important for the progression of diabetes in mice and humans^2–6^. CD8^+^ T cells are crucial for mediating direct islet killing, whereas CD4^+^ T cells may be more important for disease initiation^7,8^. Removal or complete absence of T cells in mouse models prevents diabetes, and the adoptive transfer of autoreactive T cells alone causes diabetes^9–11^. Regulatory CD4^+^ T cell (T_reg_) interactions are also pathologically culpable given that T1D donor T_regs_ exhibit decreased autologous suppressive capacity relative to HLA- and age-matched controls^12–18^. Furthermore, CD4^+^ T cells have a critical role in both augmenting CD8^+^ T cell responses and undergoing cognate interaction with B cells leading to autoantibody production^19^.

Diabetes in humans and the non-obese diabetic (NOD) mouse occurs spontaneously^1^. In NOD mice, immune cell infiltration of the pancreas first appears at approximately 4-weeks of age with the first observable immune cells surrounding the islet (peri-insulitis)^20,21^, while progression to diabetes and loss of normal glycemic control occur after 10-weeks of age^2,3^. Given that peri-insulitis dominates before intra-islet insulitis and precedes detectable loss of β cell mass by approximately 8 weeks^20^, the distribution of T cells within and outside the islet offer insight into T1D disease progression^22^. To adequately assess the spatial contributions of T cells to the autoimmune process in T1D, there is a need for quantitative imaging methodologies that are expeditious, facile and unbiased.

### Current islet image analysis approaches

Multiple imaging modalities are currently available to study the in situ distribution of both human and murine pancreatic autoreactive T cells. Among these, light microscopy visualizing hematoxylin and eosin (H&E) stained fixed/sectioned pancreas tissue is commonly used given its ability to easily distinguish normal tissue from islets with infiltrating immune cells. However, this method has multiple limitations. First, H&E staining prevents identification of immune cell subtypes. Second, H&E microscopy relies upon investigator-assigned insulitis scores that classify islet infiltration into one of five groups based upon the perceived proportion of the islet destroyed^8^. Although analysis of insulitis scores assigned to each islet allows for the detection of large differences in islet inflammation, more subtle variations can be challenging to identify. Third, visually assessing the proportion of an islet destroyed by inflammation requires the analyzer to reliably determine precise borders between the islet and surrounding pancreatic exocrine tissue, which becomes more difficult as infiltration progresses and islets are further destroyed. In older NOD mice, some islets visualized as “insulitis-free” may in fact represent late stage pathology of post-insulitic “pseudo-islets” containing only non-β endocrine cells after all beta cells have been targeted and destroyed^8^. An alternative imaging approach uses fluorescent antibody staining of specific cell subsets to visualize immune cell infiltrate using epifluorescent wide-field or confocal microscopy. These fluorescent microscopy techniques provide data that can be analyzed with traditional insulitis scoring procedures, much like H&E qualitative analyses described above^8^. In addition, pancreas sections can be evaluated by quantifying fluorescent signals, but current strategies require cumbersome manual analysis or utilize sophisticated imaging software that can be prohibitively expensive. Currently, no unbiased analytical approach exists to rapidly examine pancreatic islet immune infiltration based on accurate determination of conspicuous islet borders without the use of advanced imaging software.

### Convex hull methodology

In this current study, we introduce an automated and largely unbiased analytical technique for the efficient quantification of immune cell infiltrate and peri-insulitis with a focus on CD4^+^ and CD8^+^ T cells. Given that murine islets possess glucagon-secreting α cells that predominantly encircle the islet perimeter and are not specifically targeted by the autoimmune response^23^, our approach uses glucagon staining to better detect islet borders in islets that have already undergone β cell destruction. We demonstrate that non-infiltrated islets can be precisely modeled by overlaying minimal area convex polygons (convex hulls) that entirely capture the sum of insulin (β cell) and glucagon (α cell) signals to distinguish individual contributions of immune cell subtypes during inflammation either within or outside the islet border. By performing this entire analysis using the freeware software ImageJ equipped with the Fiji Is Just ImageJ (FIJI) image processing package^24,25^, our approach utilizes a macro that is accurate, reliable, practical and free. We suggest that identifying the location of immune infiltrate relative to the islet boundary may have implications regarding the progression of disease, the efficacy of diabetes-ameliorating therapeutics and the ability of immune cells to traffic to the site of autoimmune invasion. Importantly, this methodology can be easily adapted to other immunological markers that identify immune cells beyond T cell subsets in other autoimmune diseases or at other tissue sites.

## Results

### Macro development and image analysis:

#### Overview

**Figure 1:**
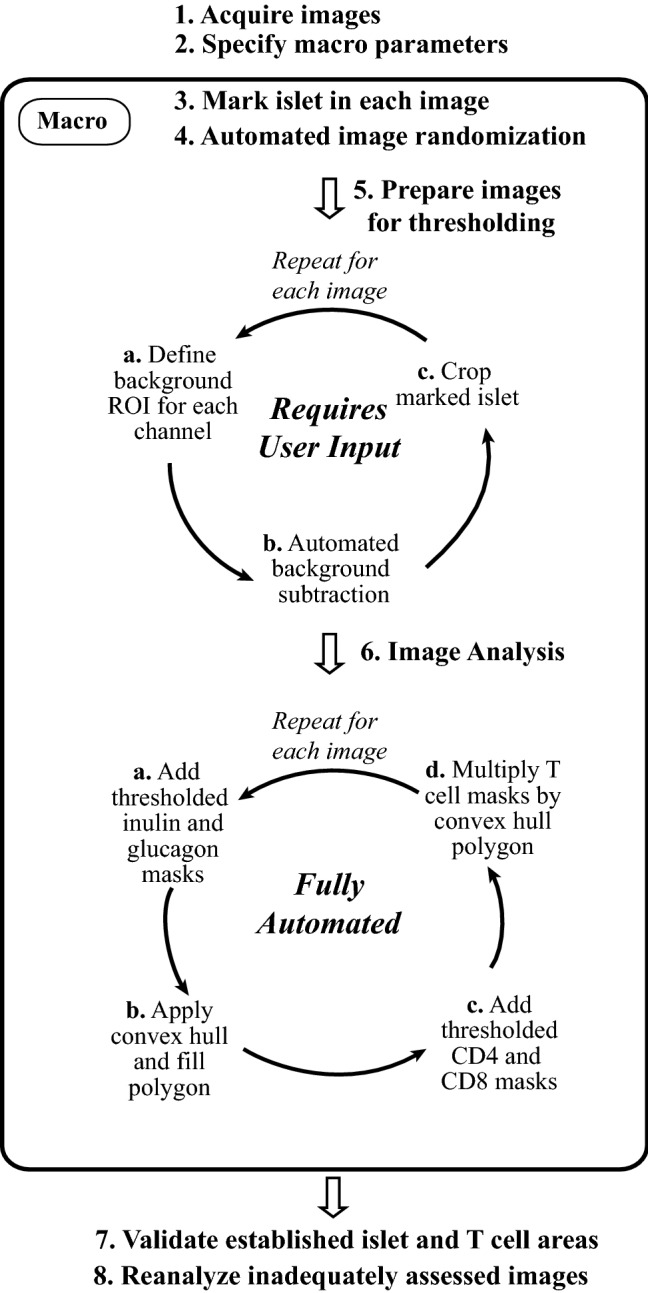
Quantitative imaging workflow for islet analysis. This step-by-step process integrates islet analysis using a FIJI-driven automated macro. After acquiring images and defining dataset-specific macro parameters, the FIJI macro guides the user through image randomization, background correction and cropping for each individual islet present in an image. Fully automated image analysis then proceeds to establish islet outlines by applying convex hulls to thresholded insulin and glucagon masks, and CD4^+^ and CD8^+^ T cell inflammation is quantified relative to the islet border to calculate insulitis (within islet border) and peri-insulitis (beyond islet border) measurements.

Before demonstrating application of our convex hull approach to determine inflammation in NOD islets through T1D progression, we first present an overview of the macro to show its accessibility. The quantitative imaging workflow contains several steps that utilize both user-driven and fully-automated approaches, depending on the requirements of each individual step (Fig. 1). Each step is described in detail in the subsequent sections. Briefly, the user first acquires epifluorescent single-channel images for each fluorophore (step 1). Next, several parameters must be set (step 2); these include specifying the cell marker for each channel, establishing thresholding and background subtraction values, and specifying the minimum pixel radius of fluorescent signal to include as foreground. After these initialization steps, the macro displays each overlaid image for the user to mark the islet-of-interest with an arrow (step 3). All images are then randomized and sequentially presented for threshold preparation (step 4) to a blinded analyzer. Because this macro cannot differentiate multiple islets in an image, images with multiple islets must be duplicated prior to analysis for individual assessment as described.

Islet images are next thresholded to create binary renderings for automated quantitative analysis. Threshold preparation requires a series of sub-steps to subtract background based on user-drawn regions of interest (ROI’s), with subsequent cropping of each islet-of-interest to ensure the correct islet is analyzed in multi-islet images (step 5). This process ensures that images with multiple islets may be analyzed, with each islet assessed independently. The bulk of the analysis is performed next, with sub-steps dedicated to first establishing the islet outline based on the combined thresholded insulin and glucagon images and then determining the amount of CD4^+^ and CD8^+^ T cell signal that falls within or outside of the newly created islet border (step 6). If background ROI’s are not drawn appropriately during step 5, unintentional user-driven errors can result in which too much background signal or too little foreground signal is included. This can cause a misrepresentation of the islet area or degree of insulitis. To mitigate this problem, a validation step is included in which representations of the calculated islet outlines and T cell signals are projected onto the original images and displayed for rapid confirmation of analytical quality (step 7). Any islets that have been inappropriately analyzed can be corrected by repeating the algorithm to improve quantification accuracy (step 8).

**Figure 2:**
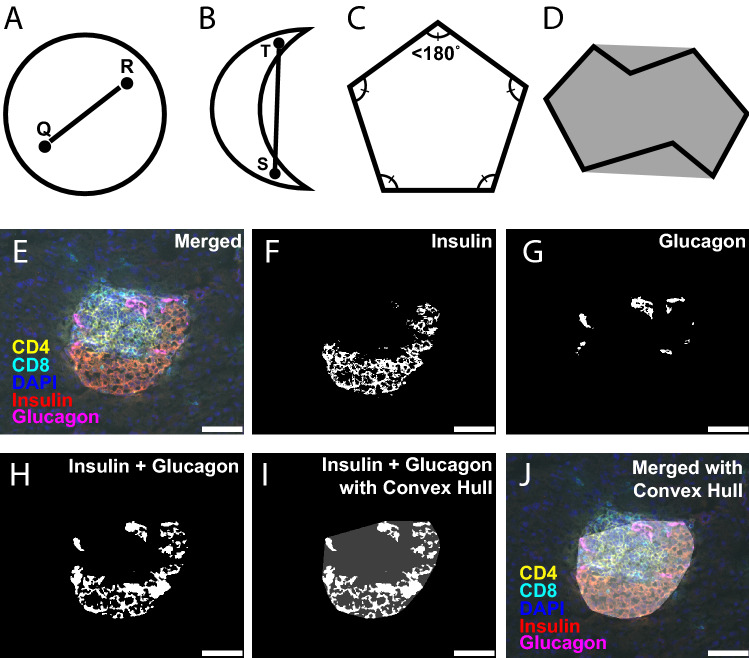
Inflamed islet borders can be defined using insulin, glucagon and a convex hull based geometrical approach. Convex hulls are two-dimensional convex polygons that can model cross-sectional images of pancreatic islets. (**A**) A circle is an example of an entirely convex shape since all segments of a straight line connecting any two randomly selected points (Q, R) within the shape lie inside the shape borders. (**B**) A crescent is an example of a shape that is not entirely convex because a straight line connecting the two points (S, T) exists outside the shape borders. (**C**) A pentagon is an example of a convex polygon because all interior angles are less than 180°. (**D**) A convex hull (grey region) is depicted to demonstrate the area that entirely encloses all points of a mixed concave/convex polygon (black outline) with the smallest possible area. (**E**–**J**) Examples of the convex hull methodology using an acquired image of an islet from a 13-week-old non-diabetic NOD mouse. (**E**) Merged image with CD4 (yellow), CD8 (cyan), DAPI (blue), insulin (red) and glucagon (magenta) staining. (**F**) Individual thresholded 8-bit insulin image. (**G**) Individual thresholded 8-bit glucagon image. (**H**) Combined insulin and glucagon images from (**F**) and (**G**). (**I**) Convex hull (transparent white) of combined insulin and glucagon images. (**J**) Original merged image with overlaid convex hull (transparent white). White bars represent 50 μm length.

A critical component of this methodology involves the application of convex hulls over the combined thresholded insulin and glucagon signals to help determine islet area (step 6b). In general, a continuous two-dimensional Euclidean plane subset may be considered convex if, given any two points selected within the confines of the space and connected by a straight line, all segments of this line lie within the Euclidean plane subset borders (Fig. 2A,B)^26^. A more intuitive description specifically defines a *polygon* as convex if all vertices have associated interior angles that are each individually less than 180°, such as a regular pentagon with equivalent interior angles of 108°. (Fig. 2C). The convex hull, then, can be described as the convex two-dimensional polygon with the smallest area that entirely encloses all data points of a Euclidean plane subset (Fig. 2D). A simple concept to visualize the convex hull is to envision a rubber band snapping tightly into place surrounding the polygon. In the case of epifluorescent imaging of pancreatic islets, the islet mass can be thought of as the relevant analyzable Euclidean plane subset with the corresponding convex hull representing the cross-sectional islet area. Importantly, thresholded insulin and glucagon single-channel images of mouse pancreatic islets contain many small, disconnected polygons due to lack of nuclear staining or dimming/elimination of signal caused by overlying infiltrated immune cells (Fig. 2E–G). Applying convex hulls to the thresholded sum of the insulin and glucagon images effectively merges these disparate signals into a single representation of islet area (Fig. 2H–J). The convex hull approach allows for improved accuracy in determining islet area over traditional visual approaches by calculating the entire area enclosed by the polygon borders. More importantly, this method can consistently establish islet borders uninfluenced by investigator interpretation that can be used to determine which T cells fall *within* the islet border (insulitis) and which fall *outside* of the islet border (peri-insulitis).

#### Background correction

**Figure 3:**
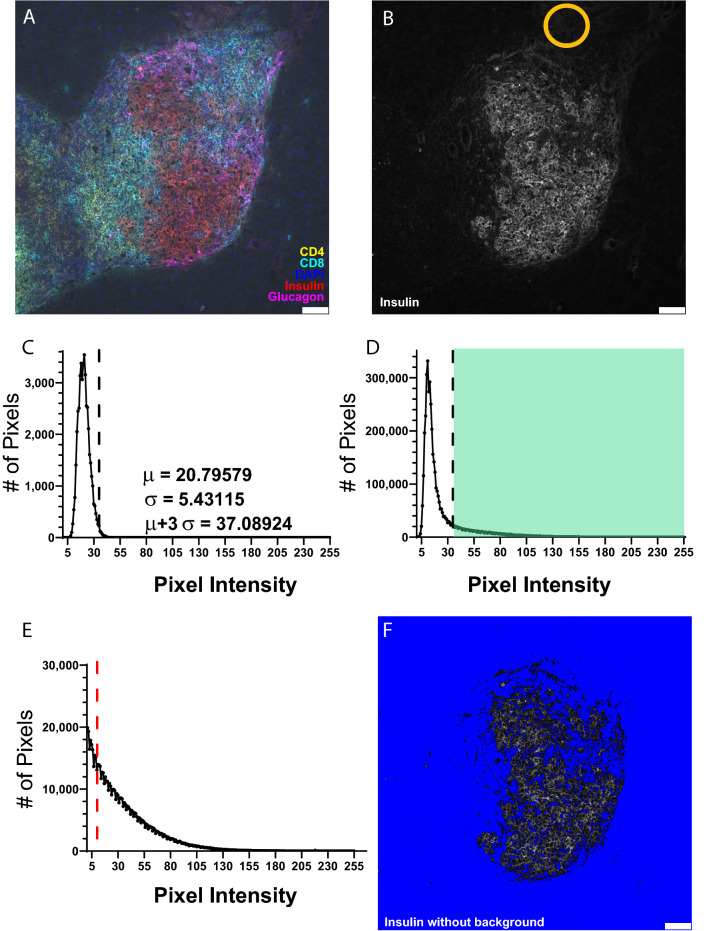
Region of interest (ROI)-based background correction allows dynamic elimination of false positive signals for individual islets. (**A**) Histology image of a 13-week-old non-diabetic NOD mouse islet with CD4 (yellow), CD8 (cyan), DAPI (blue), insulin (red) and glucagon (magenta) staining. (**B**) Individual 8-bit insulin image of islet from (**A**) with orange circle representing a valid ROI selection containing only negative background for subsequent signal subtraction. (**C**) Brightness histogram of the insulin background ROI from (**B**) demonstrating the default background subtraction value of three standard deviations (σ) above the mean (μ) (dotted line). (**D**) Brightness histogram of the entire insulin image from (**B**) depicting the subtraction value obtained from (**C**) (dotted line) with the shaded green region representing positive insulin signal and the unshaded region representing negative background signal. (**E**) Insulin brightness histogram after background subtraction with dotted red line representing an additional flexible cutoff value dependent upon the combination of fluorophores employed. (**F**) Individual 8-bit insulin image of islet from (**A**) after background subtraction with pixel values of 0 represented in blue to enhance visualization of remaining positive insulin signal. White bars represent 50 μm length.

To eliminate user bias influenced by treatment conditions when performing background correction, image randomization occurs prior to any alterations of the raw data. A blinded user first marks each islet in the image series using an arrow to ensure background correction is optimized to the XY position of the islet of interest. This step is necessary to properly identify the islet of interest in images that contain multiple islets. Image randomization proceeds using the Fisher–Yates shuffle statistical randomization algorithm to decrease any user bias for treatment groups or experimental conditions between tissues. The user then draws a small ROI containing the brightest background signal observed in each fluorescent channel to specify undesired background intensities that must be subtracted (Fig. 3A,B). Identifying an ROI with bright intensity of *only* background signal is critical; an ROI containing only dim background signal will fail to remove background regions of brighter intensity (false positives) while an ROI containing any foreground signal will remove all foreground regions of similar or lower brightness (false negatives).

The overarching goal of background correction is to minimize the proportion of false positives and negatives within an image, which is accomplished through uniform subtraction of an intensity value from all pixels in each channel. Following user-driven background ROI identification, the macro automatically selects an intensity value for each channel based on the background ROI to subtract from each pixel and removes background signal in the corresponding channel. Specifically, the macro automatically determines the mean ($\mu$) and standard deviation ($\sigma$) intensity values for the background ROI’s in each channel and calculates a “subtraction value” ($S$) using the formula $S = \mu + 3\sigma$. A properly delineated background ROI will have a brightness histogram that is approximately Gaussian, so setting $S$ three standard deviations above the mean captures > 99% of the background pixels (Fig. 3C). Notably, $S$ cannot simply be set to the maximum intensity value within the background ROI since imperceptible saturated pixels are often included in the background ROI’s, skewing the value of $S$. When considered in the context of the entire image brightness histogram for each corresponding channel, $S$ effectively functions as a delineation point to definitively separate foreground from background intensity signals (Fig. 3D). $S$ is then subtracted from each pixel in the image, “left-shifting” the brightness histogram for each channel such that background pixels are normalized to an intensity value of 0 (Fig. 3E). This process has the effect of eliminating nearly all the background signal while ensuring that all relevant foreground signals are maintained (Fig. 3F). Fine-tuning of background signal elimination occurs in later steps of image processing, though this initial background subtraction procedure accounts for the majority of background correction.

The final step in background correction involves manual cropping of each islet-of-interest and associated immune cells. In this analysis, only immune cells continuous with the insulin and glucagon signals are included as islet-associated immune cells, with all intervening tissue resident CD8^+^ T cells and other niduses of inflammation (e.g. lymphocytes occupying exocrine pancreas blood vessels) excluded. The crop region is drawn on the merged channel and automatically applied to each individual fluorescent channel to avoid quantifying signals derived from inflamed islets in the image other than the islet of interest. The background correction and cropping process is then repeated for each islet, after which the fully automated insulitis and peri-insulitis calculations can proceed.

#### Insulitis and peri-insulitis calculations

To allow image thresholding and promote consistency in units of length for images captured on microscopes with different magnifications or different charge-coupled device pixel sizes, all channels are first converted to 8-bit images and units of length are converted to pixels. 8-bit insulin, glucagon, CD4 and CD8 channels are then converted to thresholded masks within a predetermined range (default = 10–255 for all channels). To calculate the cross-sectional islet area, insulin and glucagon masks are added together and any remaining background noise is eliminated by removing continuous foreground regions smaller than a specified size (default = 5; will vary depending on pixel size and image magnification but should be set to approximate lymphocyte diameter). All background regions entirely surrounded by foreground (“background enclaves”) in the insulin/glucagon sum image are converted to foreground to account for lack of nuclear staining in images obtained from staining with antibodies specific for molecules confined to the cell surface or cytoplasm (e.g. insulin and glucagon). The convex hull of the insulin/glucagon sum image is calculated and all background within the convex hull is converted to foreground, establishing a thresholded region that matches the islet area.

As diabetes progresses, CD4^+^ and CD8^+^ T cells infiltrate and destroy the pancreatic islet β cells^27,28^. The degree of infiltration can be determined by the location of the inflammatory aggregate relative to the islet borders. CD4 and CD8 insulitis and peri-insulitis calculations are derived by comparing their signals to the islet area image. Thresholded CD4 and CD8 images are multiplied by the thresholded islet area image to isolate only the signals occupying the islet area. The total area of CD4^+^ and CD8^+^ T cells associated with each islet is also calculated, providing a reference for peri-insulitis calculations. The thresholded CD4 and CD8 images are then added together and the area calculations are repeated with the resultant image to provide an approximation of combined CD4^+^ and CD8^+^ T cell inflammation. β cell mass can be calculated from the insulin signal by converting all background enclaves to foreground to account for lack of nuclear staining.

To facilitate expeditious visual inspection of insulitis, peri-insulitis and islet area calculations, two validation images are generated for each analyzed islet and adjacently displayed as two linked image stacks. The first image stack depicts a transparent overlay of the islet area derived from the insulin and glucagon signals with the original false-color merged image for each islet. The second image stack depicts this same islet area overlay on a black background accompanied by the thresholded CD4 and CD8 signals. By generating these two adjacent image stacks, the user determines which islets (if any) must be reanalyzed due to inadequate user-defined background correction.

#### Convex hull validation

To validate the convex hull approach, we applied common planar convexity metrics to non-infiltrated pancreatic islets. Common convexity measurements of two-dimensional Euclidean plane subsets can be categorized into either area- or perimeter-based assessments^26^. Utilizing both area- and perimeter-based measurements to assess convexity is advantageous since minor alterations in the Euclidean plane subset can dramatically alter its corresponding convex hull. For example, protrusions and border irregularities can radically alter the area and perimeter respectively of the convex hull overlying an irregular, partially concave polygon (Fig. 2D). For both area- and perimeter-based measurements, ratiometric comparison of the Euclidean plane subset to its convex hull offers a simple and well-accepted method by which to assess the degree of convexity of the subset. Specifically, the area-based convexity measurement ($C_{Area}$) can be calculated using the formula $C_{Area} = \frac{{Area_{E} }}{{Area_{CH} }}$, where $Area_{E}$ and $Area_{CH}$ are the areas of the Euclidean plane subset and convex hull respectively. Similarly, a boundary-based convexity measurement can be derived based on perimeter ratios ($C_{Perimeter}$) using the formula $C_{Perimeter} = \frac{{Perimeter_{CH} }}{{Perimeter_{E} }}$, where $Perimeter_{CH}$ and $Perimeter_{E}$ are the perimeters of the convex hull and Euclidean plane subset respectively. The relative position of the convex hull measurement in either the numerator or denominator in both these formulae is due to the geometrical property of convex hulls generally possessing a larger area and smaller perimeter than their Euclidean plane subset counterparts. Consequently, measuring the convexity of Euclidean plane subsets in this manner yields a value between 0 and 1, with convexity increasing as this number approaches 1. A value of 1 for both the area- and perimeter-based measurements also suggests that the convex hull perfectly models the Euclidean plane subset, as is true for all regular polygons (e.g. equiangular triangles) as well as other perfectly convex shapes (e.g. circles and ellipses).

**Figure 4:**
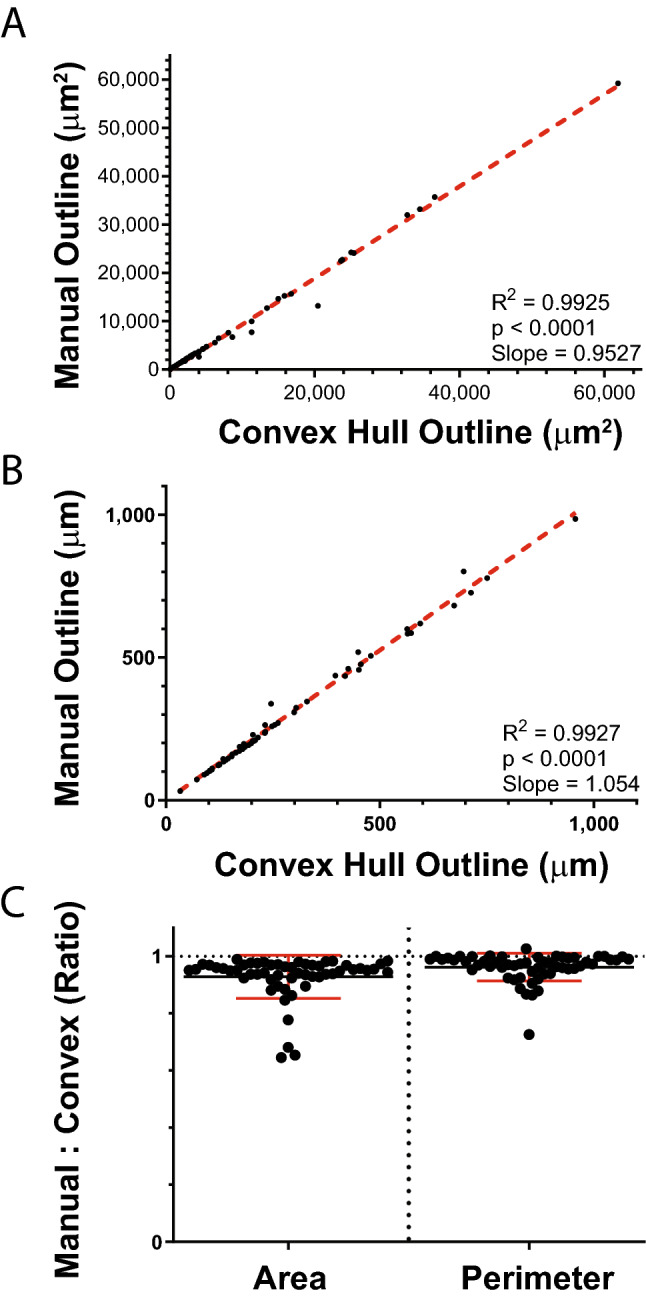
Convex hull representations of two-dimensional islet cross sections accurately model non-infiltrated islets. Islet images from 11-week-old NOD.RAG1.KO mice (n = 3) were obtained and islets (n = 55) were manually contoured by a blinded analyzer or overlaid with convex hulls derived from added insulin/glucagon signals using the macro methodology. Areas and perimeters of the cross-sectional polygons from both approaches were compared to validate the convex hull approach. (**A**,**B**) Linear regressions between (**A**) areas and (**B**) perimeters of the associated manual or convex hull outlines. (**C**) Area ratio of manual outline to convex hull outline and perimeter ratio of convex hull outline to manual outline. Dotted red lines on linear regression plots represent the line of best fit. Data are pooled from three independent experiments.

Extending these geometrical concepts to pancreatic islets, we applied the area- and perimeter-based metrics to NOD.RAG1.KO to determine the degree to which non-infiltrated cross-sectional islet areas correspond to their convex hulls. Islet outlines were first obtained by manual contouring of the overlaid images and their convex hulls were determined, with area and perimeter measurements calculated for both the original contour and the convex hull. A linear regression between the manual and convex hull areas shows a near-perfect correlation (R^2^ = 0.9925, p < 0.0001) with the convex hull areas closely matching their manual outline counterparts (m = 0.9527, 95% CI X- and Y-intercepts include 0) (Fig. 4A). Similar results were obtained with a linear regression between the manual and convex hull perimeters (R^2^ = 0.9927, p < 0.0001, m = 1.054, 95% CI X- and Y-intercepts include 0), with both the area- and perimeter-based convexity measurements average being close to 1 (Fig. 4B,C). These data suggest pancreatic islet cross sections are predominantly convex and are closely modeled by the convex hulls of their two-dimensional outlines, offering support for our method using the convex hull approach in definitively establishing conspicuous islet borders when assessing immune cell insulitis and peri-insulitis.

#### Comparison to standard qualitative approach and representation of data

**Figure 5:**
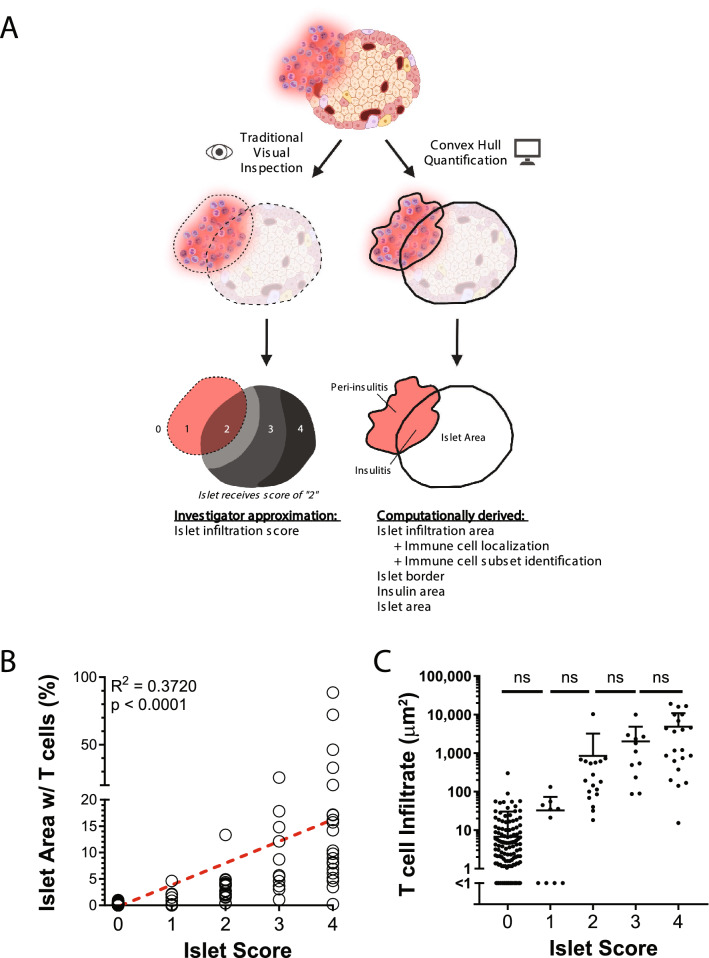
Quantitative assessment of islet infiltration detects variability that traditional qualitative islet scoring approaches fail to identify. (**A**) Diagram comparing assessment of islet infiltration using the traditional visual inspection method (left) vs. the convex hull quantification method (right). The traditional approach generates an approximate islet infiltration score (“2” for the depicted islet) while the convex hull approach provides multiple computationally derived quantifiable metrics. (**B**,**C**) Individual islet images from 4-week-old (n = 93), 13-week-old non-diabetic (n = 71) and 17-week-old diabetic NOD mice (n = 23) and 11-week-old NOD.RAG1.KO (n = 55) mice were pooled within each group (n = 3/group) and analyzed with both our quantitative methodology and a traditional qualitative islet scoring approach performed by a blinded analyzer. (**B**) Linear regression between quantified proportion of individual islet areas occupied by T cells and traditional qualitative islet scores. Dotted red line on the linear regression plot represents the line of best fit. (**C**) Qualitative islet scores for each islet plotted against their corresponding quantified T cell infiltrate areas. Islet scoring: 0—no insulitis, 1—peri-insulitis, 2—< 25% of islet mass infiltrated, 3—< 75% of islet mass infiltrated, 4—> 75% of islet mass infiltrated. Kruskal–Wallace test with post-hoc Dunn’s multiple comparison test was performed to determine statistical significance. ns, not significant. Data are pooled of three independent experiments.

Qualitative approaches to approximate the magnitude of pancreatic insulitis require visual assessment of each islet to determine an insulitis score. The range of possible scores typically represent discrete bins that correspond to the proportion of the cross-sectional islet area occupied by immune cells (Fig. 5A, left). In contrast, automated convex hull quantification generates cross-sectional area data for islet infiltration area as well as other user-defined metrics (Fig. 5A, right). Given that our quantitative method can accurately evaluate the contributions of individual immune cell subtypes to islet infiltration, we sought to directly compare our method to a conventional qualitative approach to determine if similar trends are observed. To accomplish this, we pooled cross-sectional pancreas images obtained from young, adult non-diabetic and adult diabetic NOD mice as well as adult NOD.RAG1.KO mice to obtain a broad range of islet inflammation. We next applied our quantitative macro or assigned traditional qualitative insulitis scores to each islet by a blinded assessor^8^. A moderately weak positive correlation was observed when comparing the proportion of islet area occupied by either CD4^+^ or CD8^+^ T cells determined by our quantitative method against the qualitative insulitis scores (Fig. 5B). This weak correlation was likely due to the increasing variability of T cell infiltrate for islets assigned larger insulitis scores, a phenomenon that is less problematic when islet infiltrate is quantitatively assessed (discussed below). This suggests that qualitatively binning islet infiltration by eye captures a larger range of infiltration values within each bin than that which the observer may be aware. Moreover, despite detectable differences in T cell infiltrate between the extreme ends of the insulitis scoring scale, the variability in infiltrate for each insulitis score prohibited differentiating between adjacent scores (Fig. 5C). Notably, the infiltrate proportions calculated with our quantitative method were consistently smaller than the infiltration proportions in the corresponding insulitis score bins. This may reflect an improved ability to detect only specific immune cell subsets (in this case, CD4^+^ and CD8^+^ T cells) with automated quantification or might represent differences in proportional infiltrate determination due to islet boundary disagreements between the traditional qualitative approach and the convex hull methodology. These findings confirm that quantitative assessment of islet immune cell infiltration correlates to traditional discrete qualitative evaluation but additionally provides continuous area metrics otherwise unobtainable from visual inspection analysis alone.

**Figure 6:**
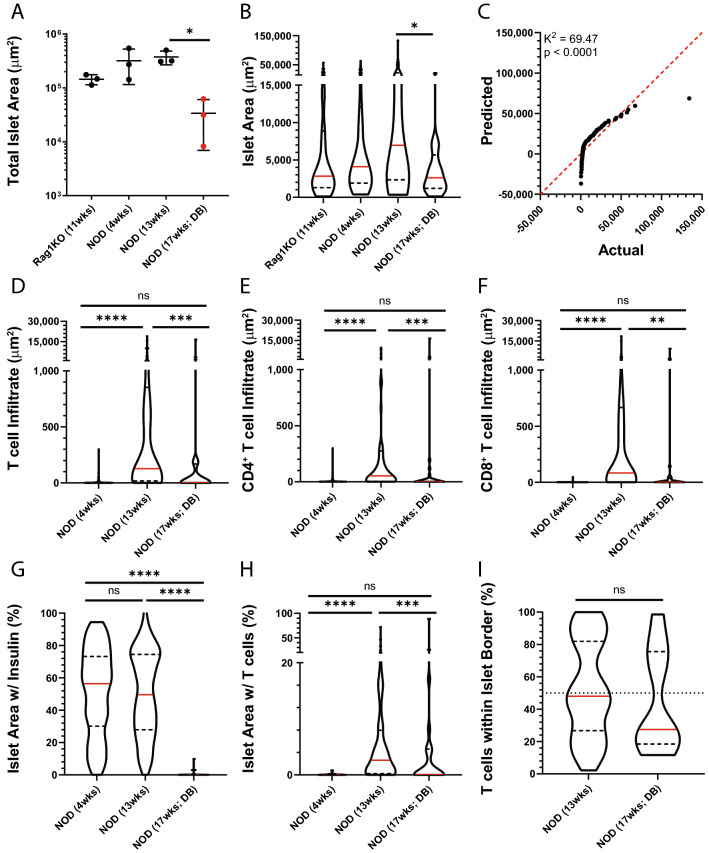
Quantitative islet analysis enables numerous metrics to assess islet inflammation throughout progression of T1D. Individual islet images from 4-week-old (n = 93), 13-week-old non-diabetic (n = 71) and 17-week-old diabetic NOD mice (n = 23) were pooled within each group (n = 3/group) and analyzed for quantification of islet area and inflammation. (**A**) Sum of total islet area for all islets imaged from each mouse. (**B**) Individual islet area from islets pooled from all mice of a given strain and age. (**C**) Quantile–quantile plot of actual individual islet areas from 13-week-old non-diabetic NOD mice, as in (**B**), compared to values predicted from a normally distributed Gaussian data set. (**D**–**F**) T cell infiltration of individual islets pooled from all mice of a given age. (**D**) T cell infiltration was assessed as combined CD4^+^ and CD8^+^ T cell infiltration as well as independent (**E**) CD4^+^ and (**F**) CD8^+^ T cell infiltration. (**G**–**I**) Metrics assessing the proportional contributions of insulin or T cell signals based on their position relative to the defined islet border of individual islets pooled from all mice of a given age. (**G**) Proportion of islet area occupied by insulin signal. (**H**) Proportion of islet area occupied by combined CD4^+^ and CD8^+^ T cell signal. (**I**) Proportion of total T cell inflammation that falls within the islet borders. Solid red lines on violin plots depict the median and dotted black lines depict the first and third quartiles. Dotted line at 50% represents a break point representing a conversion from peri-insulitis to insulitis. “DB” refers to diabetic mice. Red dots represent data points from diabetic mice. Kruskal–Wallace test with post-hoc Dunn’s multiple comparison test was performed to determine statistical significance. Data are representative of three independent experiments. *p < 0.05, **p < 0.01, ***p < 0.001, ****p < 0.0001. *ns* not significant.

To demonstrate application of this method to a data set with a broad range of islet infiltration, we independently analyzed images obtained from NOD.RAG1.KO mice and NOD mice from three age groups (young, adult non-diabetic and adult diabetic) as in Fig. 5B,C. Using this method, data obtained from analyzed islets may be summed for each mouse with each data point representing a single mouse, or single islets may be pooled from all mice in a particular group and individually plotted. In both cases, assessing islet area expectedly demonstrates a significant decrease in islet size between non-diabetic and diabetic adult NOD mice, consistent with the notion that progressive autoimmune destruction of pancreatic islets eliminates functional and observable islet mass as T1D progresses (Fig. 6A,B). Upon observing the apparent asymmetric distribution of islet area for all treatment groups (Fig. 6B), we aimed to determine whether this distribution was Gaussian to ensure selection of appropriate statistical tests. We constructed quantile–quantile (Q–Q) plots of each data set against a predicted Gaussian probability distribution to assess the variation in islet sizes relative to an expected normal bell curve. We found that the islet area in each group profoundly deviates from an expected Gaussian distribution, and this deviation from normality was observed for islet inflammation metrics as well (Fig. 6C and data not shown). Given this, we assert that data sets obtained by imaging islets are best represented with individual islets plotted and analyzed with nonparametric statistical tests such that potential alterations in data distribution and the substantial variability in islet size and inflammation between islets can be properly ascertained.

A major benefit of employing quantitative fluorescent microscopy over traditional qualitative approaches is the ability to enumerate fluorescent signals independently of one another. Given the known contributions of both CD4^+^ and CD8^+^ T cells to development of spontaneous diabetes in the NOD mouse, we hypothesized a concomitant and comparable increase in both CD4^+^ and CD8^+^ T cell infiltrate area in adult non-diabetic mice (13-weeks) relative to both young mice (4-weeks) and adult diabetic mice (17-weeks). To this end, we sought to determine the collective and individual contributions of CD4^+^ and CD8^+^ T cells to islet inflammation in our treatment groups using an unbiased and quantitative approach. Assessing bulk T cell inflammation demonstrates a significant increase in T cell infiltration from 4- to 13-weeks as autoimmune pathology advances, with a significant drop in diabetic 17-week mice (Fig. 6D). Notably, no difference in total T cell infiltrate was observed between 4-week (non-diabetic) and 17-week diabetic mice. This may reflect late-stage T1D NOD islets rapidly losing mass with subsequent death and egression of immune cells out of the pancreas as antigen becomes limiting, leading to an apparent decrease of T cells within any remaining detectable islet borders. This same trend of waxing/waning T cell infiltration as NOD mice age can also be observed for CD4^+^ and CD8^+^ T cells independently (Fig. 6E,F). Although no differences were found in the overall trend of CD4^+^ or CD8^+^ T cell infiltrate among our three selected timepoints, this finding demonstrates the ability of our method to quantitatively assess both collective and individual fluorescent signals when investigating multiple cellular subtypes.

Proportion calculations evaluating T cell inflammation and position relative to the area and borders of an islet can also be performed. One such metric assesses the proportion of insulin signal relative to islet area for each islet, which demonstrates a robust decrease for diabetic adult mice relative to non-diabetic adults and young mice (Fig. 6G). This metric can be similarly applied to T cell infiltrate as a proportion of islet area for each islet, showing a marked increase for adult non-diabetic mice compared to young mice (Fig. 6H). As with quantifying T cell infiltrate area (Fig. 6D–F), this proportion drops for adult diabetic mice to levels indistinguishable from young mice. To obtain quantitative data describing T cell position relative to islet borders, we calculated the T cell infiltrate as a proportion of total T cell signal associated with each inflamed islet for those mice that had such islets (i.e. only adult mice). Our data show no difference in T cell infiltrate as a proportion of total T cell signal between non-diabetic and diabetic adult mice, but we suggest this metric may be particularly useful for grossly defining islet inflammation as predominantly insulitis (> 50% total T cell signal occupying islet space) or peri-insulitis (< 50%) (Fig. 6I). Altogether, the data generated from this quantitative method of enumerating pancreatic islet immune cell infiltration offer multiple routes by which T cell area and relative position can be calculated.

#### Technical limitations and analytical reproducibility

**Figure 7:**
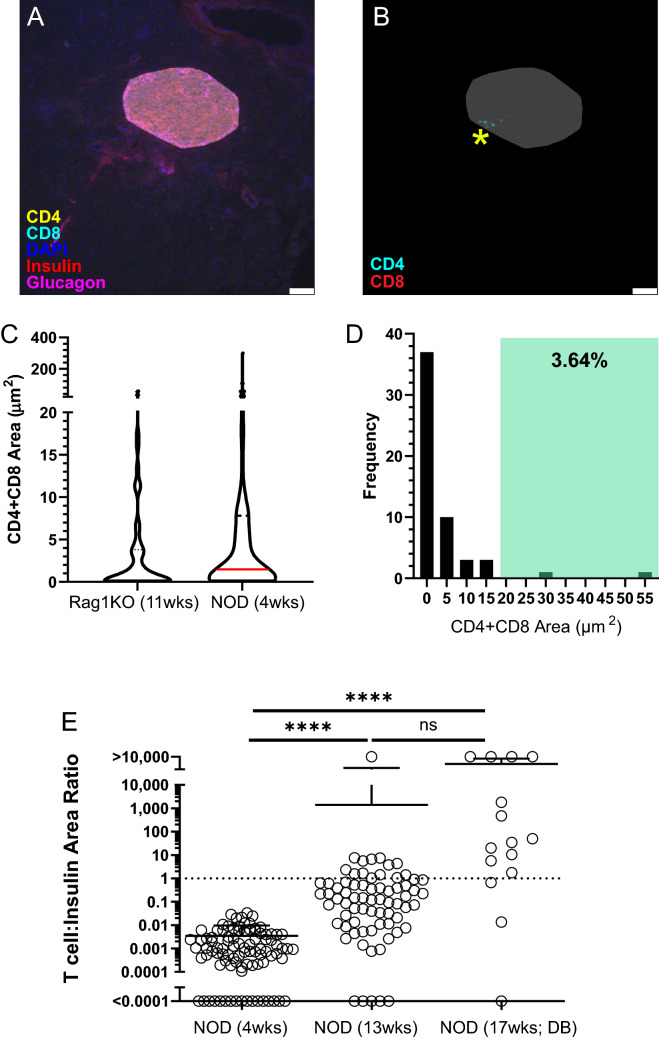
Technical limitations of quantitative islet analysis do not prevent accurate quantification of T cell inflammation. (**A**) Histology image of an 11-week-old NOD.RAG1.KO islet with CD4 (yellow), CD8 (cyan), DAPI (blue), insulin (red) and glucagon (magenta) staining with overlaid convex hull (transparent white) representing islet area. (**B**) 8-bit validation image showing background CD4 (cyan) and CD8 (red) signal on the border of the non-infiltrated islet from (**A**) (yellow star). (**C**) Total CD4^+^ and CD8^+^ T cell area detected within or near pooled islets from 11-week NOD.RAG1.KO or 4-week NOD mice (n = 3/group). (**D**) Frequency histogram of background CD4 and CD8 signals in 11-week-old NOD.RAG1.KO islets. The shaded green region depicts true positive signals above the T cell limit of detection with < 5% of non-infiltrated NOD.RAG1.KO islets showing background CD4 or CD8 signals in this range. (**E**) Ratio of T cell area to insulin area for individual islets pooled from 4-week, 13-week non-diabetic and 17-week diabetic NOD mice (n = 3/group). White bars on histology images represent 50 μm length. Solid red lines on violin plots depict the median and dotted black lines depict the first and third quartiles. “DB” refers to diabetic mice. Kruskal–Wallace test with post-hoc Dunn’s multiple comparison test was performed to determine statistical significance. Data are representative of three independent experiments. *p < 0.05, **p < 0.01, ***p < 0.001, ****p < 0.0001. *ns* not significant.

Although this quantitative imaging methodology accurately reflects islet area and T cell inflammation, there are limitations to consider. For example, despite a robust background correction protocol (described above) that avoids the need for complex spectral microscopy or linear unmixing protocols, a small amount of false positive CD4^+^ and/or CD8^+^ T cell signal appears in a minor subset of NOD.RAG1.KO islets, predominantly within the islet border (Fig. 7A). This is principally caused by spectral overlap between different fluorophores, in this case between CD4-AF594 and Insulin-Cy3 causing false positive CD4 signal within the islet borders of immunodeficient NOD.RAG1.KO mice (Fig. 7B, yellow asterisk)^29^. This effect causes non-infiltrated 11-week NOD.RAG1.KO islets to appear infiltrated at levels comparable to slightly infiltrated 4-week NOD islets despite a known absence of lymphocytes in RAG1.KO mice. (Fig. 7C). To mitigate this issue, quantified inflammation area ≤ 20 µm^2^ is designated as beyond the limit of detection with this methodology since < 5% of non-infiltrated NOD.RAG1.KO islets have false positive T cell signals > 20 µm^2^ (Fig. 7D). Given that total islet area decreases as T1D progresses (Fig. 6A,B), establishing precise islet border demarcation becomes progressively more difficult throughout disease development. For late-stage diabetic and heavily infiltrated islets, alternative metrics may be employed to assess disease severity. For example, an inverse relationship exists between total T cell area and insulin area as T1D progresses (i.e. as T cell area increases, β cells are destroyed and insulin area decreases), making a ratio of T cell area to insulin area an alternative option for determining pathological extent of disease (Fig. 7E).

**Figure 8:**
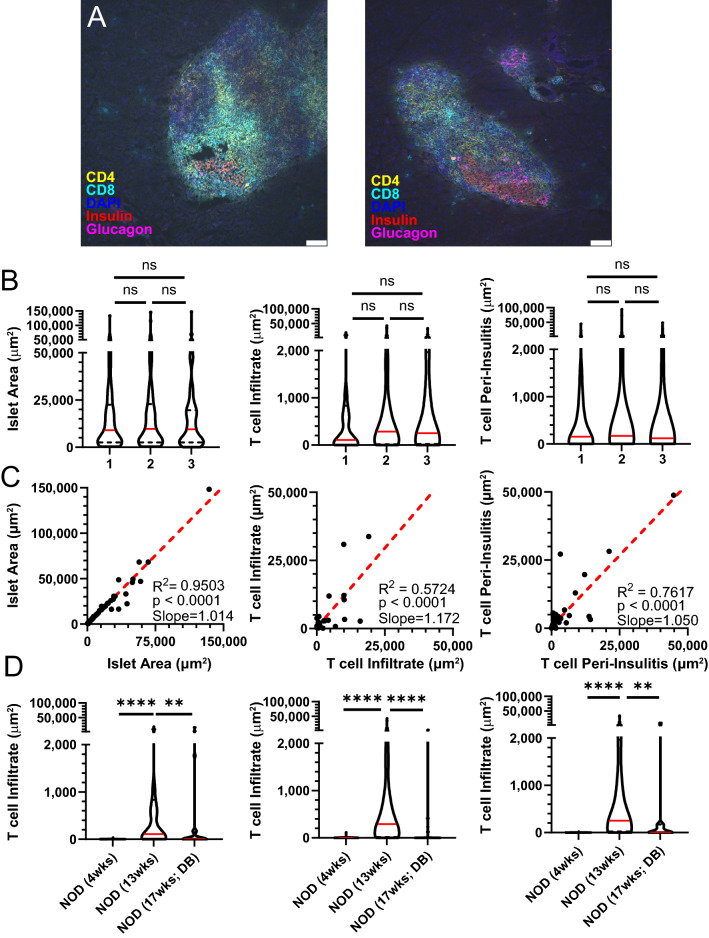
Quantitative islet analysis yields reproducible data when performed by multiple independent analyzers. (**A**) Histology images of two representative 13-week-old non-diabetic NOD mouse islets with CD4 (yellow), CD8 (cyan), DAPI (blue), insulin (red) and glucagon (magenta) staining showing heavily infiltrated islet with islet borders that are difficult to define (yellow stars). (**B**–**D**) A randomly selected subset of islet images (n = 66) obtained from 4-week, 13-week non-diabetic and 17-week diabetic NOD mice (n = 3/group) were separately assessed using the quantitative methodology by three independent and blinded analyzers. (**B**) Islet area, T cell infiltration and T cell peri-insulitis of individual islets pooled from all mice of a given age as assessed by analyzers 1–3. (**C**) Linear regressions of islet area, T cell infiltration and T cell peri-insulitis between analyzers 1 (x-axis) and 2 (y-axis). (**D**) Combined CD4^+^ and CD8^+^ T cell infiltration of different NOD mouse timepoints as assessed by analyzers 1–3. White bars on histology images represent 50 μm length. Dotted red lines on linear regression plots represent the line of best fit. Solid red lines on violin plots depict the median and dotted black lines depict the first and third quartiles. “DB” refers to diabetic mice. Kruskal–Wallace test with post-hoc Dunn’s multiple comparison test was performed to determine statistical significance. Data are representative of three independent experiments. *p < 0.05, **p < 0.01, ***p < 0.001, ****p < 0.0001. *ns* not significant.

Data reproducibility and scientific rigor between independent investigators is a crucial component of ensuring the accuracy of an experimental method or analytical technique. Although user bias is largely eliminated with this image analysis methodology, the process of ROI-based background subtraction and islet cropping notably requires user input (Fig. 1, step 5). To assess reproducibility of results between users, we randomly selected a subset of islets from 4-week, 13-week non-diabetic and 17-week diabetic NOD mice and these data were independently processed by three blinded analyzers. Results were highly reproducible at 4-weeks, when robust inflammation is not yet present and therefore definitive islet border identification is straightforward (data not shown). More variability in results occurred at 13-weeks likely due to the preponderance of heavily infiltrated islets with difficult to define borders at this timepoint (Fig. 8A, yellow stars). Despite this, there were no differences between the three analyzers regarding total islet area, T cell infiltrate, T cell peri-insulitis or any of the individual CD4^+^ or CD8^+^ T cell signals at 13-weeks (Fig. 8B and data not shown). To determine variability between users on an individual islet basis, we next performed linear regressions of each metric for every islet comparing all permutations of analyzers 1–3. We observed strong and significant correlations with 1:1 concordance between analyzers 1 and 2 (slopes ≈ 1) for all metrics assessed in Fig. 7B, and these correlations were also present between analyzers 1 and 3 and analyzers 2 and 3 at the 13-week timepoint (Fig. 8C and data not shown). Expectedly, islets with greater inflammation demonstrated a larger variability in infiltrate quantification between different analyzers, evidenced by greater deviation of islets with larger infiltrate from the line of best fit. Importantly, all three analyzers internally replicated the waxing and waning phenotype observed in Fig. 6D–F in which total T cell infiltrate is greatest at 13 weeks relative to 4-week non-diabetic and 17-week diabetic NOD mice, and this same result was obtained for isolated CD4^+^ and CD8^+^ infiltrate as well (Fig. 8D and data not shown). Taken together, these data suggest that multiple individuals employing this quantitative methodology on identical data sets will consistently achieve comparable results in a reproducible manner.

## Discussion

With this study, we introduce an automated convex hull-based analytical procedure using a macro written in FIJI for the rapid and precise identification of pancreatic islet borders and subsequent quantification of islet area, insulitis and peri-insulitis. We demonstrate the application of convex hulls to cross-sectional islet images and show the accurate modeling of non-infiltrated islets with this approach. An ROI-based background correction scheme represents one possible method to eliminate background signals, allowing us to establish that our analysis captures variability that traditional qualitative approaches neglect. We additionally show that various infiltration metrics can be obtained from our macro-derived quantification parameters and the technical limitations associated with quantifying inflammation in heavily infiltrated islets do not preclude determination of inflammatory severity. Finally, we provide evidence that this methodology is reproducible between users, even as islet T cell infiltration increases.

Modeling the cross-sectional area of pancreatic islets using the convex hull approach is advantageous for several reasons: (1) it generates a complete two-dimensional polygon that accurately estimates islet area without leaving interior signal gaps that would result from simple addition of insulin and glucagon signals (Fig. 2F, left panel); (2) it establishes continuous islet outlines in cases where immune-mediated pancreatic islet destruction has obfuscated the islet border (Fig. 2F, middle and right panels); and (3) delineation of islet borders occurs rapidly and with minimal bias when insulin and glucagon are present. However, an underlying assumption is that the areas of two-dimensional mouse pancreatic islet cross-sections are accurately modeled by overlaid convex hulls. Given that pancreatic islets are thought to be roughly spherical in shape when embedded in pancreatic exocrine tissue^30^, this assumption was reasonable. Corroborating this, our data show similar modeling of non-infiltrated islet areas by both automated convex hulls and manual contouring (Fig. 4), suggesting that convex hulls can model non-infiltrated islet areas to a high degree of accuracy.

A significant benefit of our automated convex hull T cell infiltration analysis is the inclusion of validation images produced by the macro to allow rapid user verification of analysis quality, with the opportunity to reanalyze images that had their background inappropriately corrected. To further amplify the scientific rigor of this approach, we recommend having a blinded investigator assess the quality of the validation images before proceeding to repeat quantification. For data sets with heavily infiltrated islets and an evenly distributed gradient of CD4 and CD8 intensity values that make defining a single background ROI challenging, multiple analyses may be necessary to achieve appropriate quantification of T cell inflammation. Variability can be further decreased by averaging quantified values obtained from multiple independent analyzers, effectively treating these data points as technical replicates. With these technical augmentations in mind, our methodology can reliably yield accurate results despite the necessity of user input during background correction and islet cropping.

Properly differentiating between foreground and background signals is a crucial step in quantitative fluorescent microscopy analyses to ensure that quantified signals represent specific staining. Given that non-specific staining of biological tissue sections with fluorophore-conjugated antibodies is inherently non-uniform due to topological variations in tissue architecture and differential islet inflammation, background staining intensity of a single fluorescent channel can vary across the histological landscape^29^. This problem is compounded when numerous fluorophores are used, since these local variations in staining intensity contribute to irregular fluorescent spillover or light contamination when comparing multiple positions in the two-dimensional plane. Therefore, addressing the issue of epi-fluorescent background correction requires adaptive identification of these subtle, location-dependent background staining variations rather than a static background subtraction approach applied equally and concurrently to all images. To solve this problem, background correction in our approach is performed serially on each image for each individual fluorescent channel with blinded user-driven identification of background ROI’s on which to base pixel subtraction near the islet being analyzed. Many automated approaches to background correction exist in FIJI that may be alternatively employed, but these approaches utilize algorithms that do not consistently capture the vast range of inflammation that exists amongst islets in diverse T1D disease states.

Depending on the fluorescent antibodies employed in imaging, a small amount of false positive signal is possible. As shown in Fig. 7C, the effects of spectral overlap with our fluorescent panel commonly produce background signals that occupy up to 20 µm^2^ area. Given that this spectral overlap applies to both non-infiltrated and infiltrated islets, we do not recommend reanalyzing non-infiltrated islets with small amounts of false positive T cell signals with the goal of eliminating this signal. Doing so will non-uniformly skew quantification since false positives are nearly impossible to discern in heavily infiltrated islets. Importantly, for experiments assessing islets with little immune infiltration, the limit of detection must be determined for each unique panel of fluorescent markers by assessing false positive signals on islets known to be non-infiltrated (e.g. NOD.RAG1.KO or young NOD islets). If imaging infiltrated islets (e.g. non-diabetic 13-week NOD mice) using a panel of fluorescent markers with minimal spectral overlap, it is likely not necessary to consider the limit of detection given that the fluorophores will not spill into adjacent channels.

A challenge that is common to both quantitative and qualitative methods is the difficulty in accurately establishing islet borders in late-stage diabetic and heavily infiltrated islets. Autoimmune disease necessarily involves destruction of autoantigen-associated tissue and in the case of T1D, insulin-producing β cells are targeted first with the eventual destruction of the entire islet of Langerhans. Although our quantitative approach utilizes glucagon staining of α cells to better assess islet area in islets where β cell loss has already begun, late-stage T1D islets eventually undergo complete destruction of all endocrine components, including the α cells. This means that total islet area decreases as T1D progresses (Fig. 6A,B) but more importantly, it suggests that precise islet border demarcation becomes progressively more difficult throughout disease development. Positional data regarding the distribution of immune cell subtypes relative to the islet border in mice with heavily infiltrated islets may be less reliable and other metrics to assess disease severity might be alternatively employed (Fig. 7E). Despite this, metrics that are difficult to assess (e.g. insulitis and peri-insulitis) still demonstrate significant 1:1 concordance between analyzers, even when comparing analyses with heavily infiltrated islets performed by different users (Fig. 8C; p < 0.0001 and slope ≈ 1). This suggests that even though convex hull modeling of cross-sectional pancreatic islets suffers from similar uncertainty in defining faithful inflamed islet borders to qualitative approaches, the method is capable of reliably quantifying area metrics of islet-associated CD4^+^ and CD8^+^ T cells.

Although the automated convex hull methodology for insulitis quantification has been discussed only in the context of murine T1D, this method may have applications for analysis of human samples as well. Human pancreata available for histology are exceptionally rare given the contraindication to biopsy, the rapidity of processing required post-autopsy to prevent exocrine enzymatic tissue autolysis and the improvement of T1D therapies leading to fewer available cadaveric samples^20^. Further complications include the transient nature of human insulitis that disappears soon after symptomatic onset and the lack of human α cell distribution encircling the islet periphery as in mice^20,23^, likely making convex hull approximation of late-stage diabetic human islets challenging. Nevertheless, automated quantitative analysis may be useful by employing alternative measurements in those samples that do exist, such as investigating the varying penetration depths of immune cell subtype infiltration localized to inflamed islets.

A unique advantage of the automated convex hull approach to assessing islet infiltration is the flexibility available in selecting alternative markers and metrics for islet inflammation analysis. Although CD4^+^ and CD8^+^ T cells were the only immune cells included in this study, these cell types could be further classified based on markers identifying their corresponding helper and cytotoxic subsets. B cells, dendritic cells and various innate cells may also be analyzed with the convex hull methodology. Additional measurements may also be of interest to future investigators, such as assessment of infiltrate density and distribution throughout the islet structure. Consequently, a compelling topic for future inquiry involves determining whether immune cell subtypes differentially contribute to the insulitis or peri-insulitis infiltrate regions at various stages of disease. It is reasonable to expect that the innate and adaptive arms of the immune system may interact with the islet border and interior structures differently dependent upon overall disease progression, islet architecture and other factors, and the convex hull approach provides an avenue by which this subject might be evaluated.

In summary, our automated approach to islet image analysis is particularly robust in producing quantifiable, replicable data reflecting pancreatic islet area and the location and area of immune cell subsets relative to islet borders. This method models cross-sectional pancreatic islet borders using convex hull processing to reflect islet area even as immune-mediated β cell destruction progresses. Convex hull representation of islet borders is especially accurate in islets without heavy infiltration, with late-stage diabetic islets posing a greater challenge due to autoimmune-mediated destruction of detectable islet components. Additionally, the capacity to expand this approach to include extra imaging channels corresponding to supplemental cellular markers or to apply this methodology to other tissue sites with defined architecture further augments the versatility of the method for quantifying microscopic cellular interactions.

## Methods

### Equipment and reagents

#### Epi-fluorescent microscopy

Pancreata were harvested and frozen in OCT compound (Sakura Finetek, Torrance, CA) as previously described^31,32^. Pancreata were cut into two groups of 10 sequential 7-μm-thick sections separated by a depth of 100-μm using a Leica CM1860 UV cryostat (Leica Microsystems, Buffalo Grove, IL) and mounted on glass slides (Fisherbrand ProbeOn Plus slides, 22-230-900; Thermo Fisher Scientific). Slides were fixed in cold (4 °C) acetone (L10407-AU; Thermo Fisher Scientific) for 10 min and stored at − 20 °C for no more than 3 months. For insulitis quantification, slides were first warmed to 25 °C and pancreas sections were hydrated in PBS for 10 min. Sections were then blocked with 5% bovine serum albumin (BSA, 9048-46-8; Sigma-Aldrich) in PBS for 1 h at 25 °C followed by primary antibody staining overnight at 4 °C in 5% BSA. Primary antibodies used were guinea pig anti-insulin (A0564; Dako, Carpinteria, CA) at 1:1000 and rabbit anti-glucagon (EP3070; Abcam, Cambridge, MA) at 1:5000. Pancreas sections were then stained with secondary and direct conjugate antibodies for 1 h at 25 °C in 5% BSA. Secondary antibodies used were donkey anti-guinea pig IgG (H and L chain) (Cy3, 706165148; Jackson ImmunoResearch, West Grove, PA) at 1:1000 and donkey anti-rabbit IgG (H and L chain) (Alexa Fluor 647, 711605152; Jackson ImmunoResearch, West Grove, PA), and direct conjugates used were anti-CD4 (Alexa Fluor 594, GK1.5; BioLegend) at 1:50 and anti-CD8 (Brilliant Violet 480, 53-6.7; Thermo Fisher Scientific) at 1:200. Slides were mounted with ProlongGold antifade reagent with DAPI (P36935; Thermo Fisher Scientific) using Gold Seal cover slips (12-518-108A; Thermo Fisher Scientific). Images were acquired on a Leica DM6000B epifluorescent microscope with a 20 × objective (Leica Microsystems, Buffalo Grove, IL). Islet scoring was performed by a blinded investigator using the following scale: 0, no insulitis; 1, peri-insulitis; 2, 25% of islet mass infiltrated; 3, 75% of islet mass infiltrated; 4, more than 75% of islet mass infiltrated^8^.

#### Mice

Female NOD (Taconic) and NOD.RAG1.KO (Jackson Laboratories) mice were housed in specific pathogen–free conditions. The Institutional Animal Care and Use Committee of the University of Minnesota (IACUC-UMN) approved all animal experiments. All animal work was conducted in accordance with the guidelines and regulations imposed by the IACUC-UMN and all reporting in this manuscript follows the Animal Research: Reporting of In Vivo Experiments (ARRIVE) guideline recommendations^33^.

#### Assessment of diabetes

Blood glucose concentrations were measured from female NOD mice with Contour glucose meters with associated Contour test strips (Bayer). Mice were designated diabetic with two consecutive readings of > 250 mg/dL.

#### Image analysis and macro development

All image analyses were performed using ImageJ equipped with the Fiji Is Just ImageJ (FIJI) image processing package^24,25^. Automation of ImageJ commands was executed using the IJ1 Macro language and code was written with the ImageJ macro processing plugin (Supplementary data). All processing parameters (e.g. thresholding) were applied uniformly to all pixels within an image.

## Supplementary Information

Supplementary Information.

## Data Availability

The macro employed in this study is available in supplementary material. Additionally, any data generated in this study are freely available upon request.
