# Supplementary material for: Enhanced CD4+ and CD8+ T cell infiltrate within convex hull defined pancreatic islet borders as autoimmune diabetes progresses
Source: Sci Rep. 2021 Aug 25;11:17142. doi: 10.1038/s41598-021-96327-2 (PMC8387412; doi:10.1038/s41598-021-96327-2)
Supplement: Supplementary file 1 — Supplementary Information. [file 41598_2021_96327_MOESM1_ESM.pdf]

Supplementary data

```
/*
The text below up until "MACRO STARTS HERE" are adjustable parameters for variables found throughout the macro.
"subimage" refers to the five channels. Channel order matters and "subimage1" must be "overlay". "SD" is the # of standard deviations above the mean background ROI brightness to be subtracted from the channel.
"LowerThreshold" and "UpperThreshold" refers to the minimum and maximum brightness values respectively for thresholding each channel.
"Islet_Outlier_Size" refers to the minimum background pixel diameter removed from the combined insulin and glucagon masks prior to convex hull application.
"Arrow" parameters refer to the settings that govern the appearance of the arrows used for islet identification.
*/
subimage1 = "overlay";
subimage2 = "CD8";
	subimage2_SD = 3;
subimage3 = "insulin";
	subimage3_SD = 3;
subimage4 = "CD4";
	subimage4_SD = 3;
subimage5 = "glucagon";
	subimage5_SD = 3;

LowerThreshold_CD4 = 10;
UpperThreshold_CD4 = 255;
LowerThreshold_CD8 = 10;
UpperThreshold_CD8 = 255;
LowerThreshold_Insulin = 10;
UpperThreshold_Insulin = 255;
LowerThreshold_Glucagon = 10;
UpperThreshold_Glucagon = 255;

Islet_Outlier_Size = 5;

Arrow_Width = 3
Arrow_Size = 10
Arrow_Color = "White"
Arrow_Style = "Filled"
```

```
//MACRO STARTS HERE

//Establishes measurement/arrow parameters and specifies input/output directories
run("Set Measurements...", "area mean standard redirect=None decimal=5");
run("Arrow Tool...", "width=Arrow_Width size=Arrow_Size color=Arrow_Color style=Arrow_Style");
path_input = getDirectory("Input Folder Directory");
path_output = getDirectory("Output Folder Directory");

//Identifies the total number of islets to analyze, "n"
run("Image Sequence...", "open=path_input sort");
name = getTitle();
n = nSlices/5;
run("Stack Splitter", "number=n");
close(name);

//Establishes an array to assign a unique name to each open stack (each stack corresponds to an islet to analyze)
stackIDs=newArray(nImages);
for (i=1; i<=nImages; i++) {
    selectImage(i);
    stackIDs[i-1]=getImageID();
}

setTool("arrow");

//User assigns an arrow to each islet to ensure the appropriate islet is analyzed in multi-islet images after image randomization
for (i=0; i<=stackIDs.length-1; i++){
        selectImage(stackIDs[i]);
        waitForUser("Use the Arrow tool to identify islet to analyze in this image, \nthen click 'OK' to proceed.");
        run("Add Selection...");
}

//Fisher-Yates Shuffle to randomize images
function shuffle(array) {
   p = array.length;
   while (p > 1) {
      k = randomInt(p);
      p--;
      temp = array[p];
```

```
      array[p] = array[k];
      array[k] = temp;
   }
}

function randomInt(p) {
   return p * random();
}

shuffle(stackIDs);

setTool("freehand");

//Background correction and islet cropping
for (i=0; i<stackIDs.length; i++){
        //Positions windows so active channels are visible
        selectImage(stackIDs[i]);
        title = getTitle();
        rename("Current_Overlay");
        run("Make Substack...", "delete slices=2-5");
        selectWindow("Current_Overlay");
        setLocation(0, 0, 800, 800);
        selectWindow("Substack (2-5)");
        setLocation(800, 0, 800, 800);

        //User-driven ROI-based background correction for each channel
        setSlice(1);
        run("Brightness/Contrast...");
        selectWindow("B&C");
        setLocation(1550,250);
        waitForUser("Outline brightest region of negative " + subimage2 + " signal using the ROI tool,\nthen click 'OK' to proceed.");
        run("Measure");
        background1 = (getResult("Mean", 0) + (subimage2_SD * getResult("StdDev", 0)));
        run("Select All");
        run("Subtract...", "value=background1");
        run("Clear Results");
        close("Results");
```

```
setSlice(2);
waitForUser("Outline brightest region of negative " + subimage3 + " signal using the ROI tool,\nthen click 'OK' to proceed.");
run("Measure");
background2 = (getResult("Mean", 0) + (subimage3_SD * getResult("StdDev", 0)));
run("Select All");
run("Subtract...", "value=background2");
run("Clear Results");
close("Results");

setSlice(3);
waitForUser("Outline brightest region of negative " + subimage4 + " signal using the ROI tool,\nthen click 'OK' to proceed.");
run("Measure");
background3 = (getResult("Mean", 0) + (subimage4_SD * getResult("StdDev", 0)));
run("Select All");
run("Subtract...", "value=background3");
run("Clear Results");
close("Results");

setSlice(4);
waitForUser("Outline brightest region of negative " + subimage5 + " signal using the ROI tool,\nthen click 'OK' to proceed.");
run("Measure");
background4 = (getResult("Mean", 0) + (subimage5_SD * getResult("StdDev", 0)));
run("Select All");
run("Subtract...", "value=background4");
run("Clear Results");
close("Results");

//Recombines channels into a single stack
selectWindow("Current_Overlay");
run("To ROI Manager");
run("Concatenate...", "  title=Current_Image image1=Current_Overlay image2=[Substack (2-5)]");

//Crops islet and cleans up unused open windows
setSlice(1);
run("From ROI Manager");
roiManager("Show All without labels");
selectWindow("ROI Manager");
setLocation(400, 0);
```

```
	selectWindow("Current_Image");
	setLocation(400, 0, 800, 800);
	selectWindow("B&C");
	close("B&C");
	waitForUser("Crop islet using the ROI tool, \nthen click 'OK' to proceed");
	setSlice(2);
	run("Clear Outside", "slice");
	setSlice(3);
	run("Clear Outside", "slice");
	setSlice(4);
	run("Clear Outside", "slice");
	setSlice(5);
	run("Clear Outside", "slice");
	setSlice(1);
	run("Select None");
	run("Remove Overlay");
	roiManager("Deselect");
	roiManager("Delete");
	close("ROI Manager");
	selectWindow("Current_Image");
	rename(title);
}

//Fully-automated image analysis to establish islet borders and quantify inflammation
for (i=1; i<=n; i++){
	//Renames each channel with the appropriate marker
	run("Stack to Images");
	rename(subimage5);
	run("Put Behind [tab]");
	rename(subimage4);
	run("Put Behind [tab]");
	rename(subimage3);
	run("Put Behind [tab]");
	rename(subimage2);
	run("Put Behind [tab]");
	Title = getTitle();
		Title = replace(Title, ".tif", "");
		Title = replace(Title, ".tiff", "");
```

```
	rename(subimage1);

	//Thresholds insulin and glucagon channels
	selectWindow("insulin");
	setOption("ScaleConversions", true);
	run("8-bit");
	run("Properties...", "channels=1 slices=1 frames=1 unit=pixels pixel_width=1 pixel_height=1 voxel_depth=1.0000000");
	selectWindow("glucagon");
	setOption("ScaleConversions", true);
	run("8-bit");
	run("Properties...", "channels=1 slices=1 frames=1 unit=pixels pixel_width=1 pixel_height=1 voxel_depth=1.0000000");
	selectWindow("insulin");
	setThreshold(LowerThreshold_Insulin, UpperThreshold_Insulin);
	setOption("BlackBackground", true);
	run("Convert to Mask");
	selectWindow("glucagon");
	setThreshold(LowerThreshold_Glucagon, UpperThreshold_Glucagon);
	setOption("BlackBackground", true);
	run("Convert to Mask");

	//Combines insulin and glucagon channels and cleans up background/foreground signals.
	imageCalculator("Add create", "insulin","glucagon");
	selectWindow("Result of insulin");
	run("Remove Outliers...", "radius=Islet_Outlier_Size threshold=1 which=Bright");
	selectWindow("Result of insulin");
	run("Fill Holes");

	//Determines the average pixel value of the combined and thresholded insulin and glucagon channels. This is necessary as a value of "0" here prevents subsequent analysis.
	selectWindow("Result of insulin");
	run("Measure");
	Outline_Mean = getResult("Mean", 0);
	run("Clear Results");
	close("Results");

	//If a signal was derived from the combined and thresholded glucagon and insulin channels, analysis can proceed.
	if (Outline_Mean > 0){
		//Applies convex hull to combined and threhsolded glucagon and insulin channels
```

```
run("Create Selection");
run("Convex Hull");
run("Create Mask");
selectWindow("Result of insulin");
run("Select None");

//Thresholds CD4 and CD8 channels
selectWindow("CD4");
run("8-bit");
run("Properties...", "channels=1 slices=1 frames=1 unit=pixels pixel_width=1 pixel_height=1 voxel_depth=1.0000000");
setThreshold(LowerThreshold_CD4, UpperThreshold_CD4);
setOption("BlackBackground", true);
run("Convert to Mask");
imageCalculator("Multiply create", "CD4", "Mask");
selectWindow("CD8");
run("8-bit");
run("Properties...", "channels=1 slices=1 frames=1 unit=pixels pixel_width=1 pixel_height=1 voxel_depth=1.0000000");
setThreshold(LowerThreshold_CD8, UpperThreshold_CD8);
setOption("BlackBackground", true);
run("Convert to Mask");
imageCalculator("Multiply create","CD8", "Mask");

//Combines inflammation channels with islet outline to create infiltration images and rename each window to reflect its subsequent quantitative measurement
selectWindow("Mask");
rename("Islet_Area");
selectWindow("CD4");
rename("CD4_Total_Area");
selectWindow("CD8");
rename("CD8_Total_Area");
selectWindow("Result of CD4");
rename("CD4_Infiltrate");
selectWindow("Result of CD8");
rename("CD8_Infiltrate");
imageCalculator("Add create", "CD4_Total_Area","CD8_Total_Area");
selectWindow("Result of CD4_Total_Area");
rename("CD4+CD8_Total_Area");
imageCalculator("Multiply create", "CD4+CD8_Total_Area","Islet_Area");
selectWindow("Result of CD4+CD8_Total_Area");
```

```
rename("CD4+CD8_Infiltrate");
imageCalculator("Multiply create", "CD4_Total_Area","CD8_Total_Area");
selectWindow("Result of CD4_Total_Area");
rename("CD4+CD8_Colocalization_Total_Area");
imageCalculator("Multiply create", "CD4+CD8_Colocalization_Total_Area","Islet_Area");
selectWindow("Result of CD4+CD8_Colocalization_Total_Area");
rename("CD4+CD8_Colocalization_Infiltrate");
selectWindow("insulin");
run("Dilate");
run("Fill Holes");
run("Erode");
rename("Insulin_Area");

//Quantifies inflammation parameters
selectWindow("Islet_Area");
run("Analyze Particles...", "summarize");
selectWindow("Insulin_Area");
run("Analyze Particles...", "summarize");
selectWindow("CD4+CD8_Total_Area");
run("Analyze Particles...", "summarize");
selectWindow("CD4+CD8_Colocalization_Total_Area");
run("Analyze Particles...", "summarize");
selectWindow("CD4_Total_Area");
run("Analyze Particles...", "summarize");
selectWindow("CD8_Total_Area");
run("Analyze Particles...", "summarize");
selectWindow("CD4+CD8_Infiltrate");
run("Analyze Particles...", "summarize");
selectWindow("CD4+CD8_Colocalization_Infiltrate");
run("Analyze Particles...", "summarize");
selectWindow("CD4_Infiltrate");
run("Analyze Particles...", "summarize");
selectWindow("CD8_Infiltrate");
run("Analyze Particles...", "summarize");
saveAs("Results", path_output+Title+"_Results");

//Creates validation images and closes unused windows
selectWindow("overlay");
```

```
		run("Add Image...", "image=Islet_Area x=0 y=0 opacity=25");
		rename("Islet_Overlay");
		run("Merge Channels...", "c1=CD8_Total_Area c5=CD4_Total_Area");
		selectWindow("RGB");
		run("Add Image...", "image=Islet_Area x=0 y=0 opacity=25");
		rename("CD4+CD8_Overlay");
		close("Islet_Area");
		close("CD4+CD8_Total_Area");
		close("CD4+CD8_Colocalization_Total_Area");
		close("CD4+CD8_Infiltrate");
		close("CD4+CD8_Colocalization_Infiltrate");
		close("CD4_Infiltrate");
		close("CD8_Infiltrate");
		close("Result of insulin");
		close("glucagon");
		close("Insulin_Area");
		selectWindow("Islet_Overlay");
		run("Flatten");
		saveAs("tiff",path_output+Title+"_Islet_Overlay");
		close();
		selectWindow("CD4+CD8_Overlay");
		run("Flatten");
		saveAs("tiff",path_output+Title+"_CD4+CD8_Overlay");
		close();
		close("Islet_Overlay");
		close("CD4+CD8_Overlay");
	}

	//If a signal was not derived from the combined and thresholded glucagon and insulin channels, analysis must be halted.
	else{
		print("Could not identify islet signal for " + Title + ". Skipping this image.");
		close("Result of insulin");
		close("glucagon");
		close("insulin");
		close("CD8");
		close("CD4");
		close("overlay");
		continue;
```

```
		}

	}

//Closes all open windows except for the "Log" window (kept open to notify user of any errors encountered when running the macro)
windowlist = getList("window.titles");
for (i=0; i<windowlist.length; i++){
	win_name = windowlist[i];
	selectWindow(win_name);
		if (win_name == "Log"){
		}
		else{
			run("Close");
		}
}

//Creates a stack of validation images and positions "Islet_Overlay" and "CD4+CD8_Overlay" images of the same islet adjacent to one another
outputlist = getFileList(path_output);
L = outputlist.length/3;
run("Image Sequence...", "open=path_output number=L starting=1 increment=3 sort");
rename("CD4+CD8_Overlay_Stack");
run("Image Sequence...", "open=path_output number=L starting=2 increment=3 sort");
rename("Islet_Overlay_Stack");
run("Combine...", "stack1=Islet_Overlay_Stack stack2=CD4+CD8_Overlay_Stack");
saveAs("tiff",path_output+"Mask Validation");

//Ensures that the "Log" window is the front window so the user can quickly identify macro errors
Windowlist = getList("window.titles");
if (Windowlist.length==0){
}
else{
	selectWindow("Log");
}
```
